# Supplementary material for: Effect of Different Physical Therapy Interventions on Brain-Derived Neurotrophic Factor Levels in Chronic Musculoskeletal Pain Patients: A Systematic Review
Source: Life (Basel). 2023 Jan 5;13(1):163. doi: 10.3390/life13010163 (PMC9867147; doi:10.3390/life13010163)
Supplement: Supplementary file 1 [file life-13-00163-s001.zip › life-2048255-supplementary.pdf]

Seach strategy

Pubmed:

Search: (((((((((((((((Pain) OR (Chronic pain)) OR (musculoskeletal pain)) OR (musculoskeletal disorder)) OR (fibromyalgia)) OR (chronic fatigue syndrome)) OR (osteoarthritis)) OR (headache disorders)) OR (central sensitization)) OR (descending inhibition)) OR (long-term potentiation)) OR (pain threshold)) OR (pain sensitivity)) OR (pain tolerance)) OR (pain perception)) AND (((((((((((((((((((((physical therapy modalities) OR (physical therapy)) OR (physiotherapy)) OR (rehabilitation)) OR (exercise)) OR (pain education)) OR (pain neuroscience education)) OR (motor imagery)) OR (guided imagery)) OR (mental practice)) OR (mental imagery)) OR (Electrotherapy)) OR (electrical stimulation)) OR (tdcs)) OR (transcranial direct current stimulation)) OR (tms)) OR (transcranial magnetic stimulation)) OR (tens)) OR (pens)) OR (dry needling))) AND ((bdnf) OR (brain derived neurotrophic factor)) Filters: Clinical Trial, Humans

APA PsycInfo

(P):
pain [mesh] OR chronic pain OR (musculoskeletal pain or musculoskeletal disorders) OR fibromyalgia OR chronic fatigue syndrome OR osteoarthritis OR headache disorders OR central sensitization OR descending inhibition OR long term potentiation OR (pain threshold or pain sensitivity or pain tolerance) OR pain perception

(I):
physical therapy modalities [mesh] OR rehabilitation [mesh] OR exercise [mesh] OR (physical therapy or physiotherapy) OR exercise therapy OR (pain education or pain neuroscience education) OR (motor imagery or guided imagery or mental practice) OR (electrotherapy or electrical stimulation) OR (tdcs or transcranial direct current stimulation) OR (tms or transcranial magnetic stimulation) OR tens OR pens OR (dry needling)

(O):
bdnf OR brain derived neurotrophic factor

MEDLINE Complete

(((((((((((((((Pain) OR (Chronic pain)) OR (musculoskeletal pain)) OR (musculoskeletal disorder)) OR (fibromyalgia)) OR (chronic fatigue syndrome)) OR (osteoarthritis)) OR (headache disorders)) OR (central sensitization)) OR (descending inhibition)) OR (long-term potentiation)) OR (pain threshold)) OR (pain sensitivity)) OR (pain tolerance)) OR (pain perception)) AND (((((((((((((((((((((physical therapy modalities) OR (physical therapy)) OR (physiotherapy)) OR (rehabilitation)) OR (exercise)) OR (pain education)) OR (pain neuroscience education)) OR (motor imagery)) OR (guided imagery)) OR (mental practice)) OR (mental imagery)) OR (Electrotherapy)) OR (electrical stimulation)) OR (tdcs)) OR (transcranial direct current stimulation)) OR (tms)) OR (transcranial magnetic stimulation)) OR (tens)) OR (pens)) OR (dry needling))) AND ((bdnf) OR (brain derived neurotrophic factor)) Filters: Clinical Trial, Humans

Scopus

(TITLE-ABS-KEY(pain) OR TITLE-ABS-KEY(chronic AND pain) OR TITLE-ABS-KEY(musculoskeletal AND pain) OR TITLE-ABS-KEY(musculoskeletal AND disorder) OR TITLE-ABS-KEY(fibromyalgia) OR TITLE-ABS-KEY(chronic AND fatigue AND syndrome) OR TITLE-ABS-KEY(osteoarthritis) OR TITLE-ABS-KEY(headache AND disorders) OR TITLE-ABS-KEY(central AND sensitization) OR TITLE-ABS-KEY(descending AND inhibition) OR TITLE-ABS-KEY(long AND term AND potentiation) OR TITLE-ABS-KEY(pain AND threshold) OR TITLE-ABS-KEY(pain AND sensitivity) OR TITLE-ABS-KEY(pain AND tolerance) OR TITLE-ABS-KEY(pain AND perception) AND TITLE-ABS-KEY(physical therapy modalities) OR TITLE-ABS-KEY(physical therapy) OR TITLE-ABS-KEY(physiotherapy) OR TITLE-ABS-KEY(rehabilitation) OR TITLE-ABS-KEY(exercise) OR TITLE-ABS-KEY(pain education) OR TITLE-ABS-KEY(pain neuroscience education) OR TITLE-ABS-KEY(motor imagery) OR TITLE-ABS-KEY(guided imagery) OR TITLE-ABS-KEY(mental practice) OR TITLE-ABS-KEY(mental imagery) OR TITLE-ABS-KEY(Electrotherapy) OR TITLE-ABS-KEY(electrical stimulation) OR TITLE-ABS-KEY(transcranial direct current stimulation) OR TITLE-ABS-KEY(tdcs) OR TITLE-ABS-KEY(transcranial magnetic stimulation) OR TITLE-ABS-KEY(tms) OR TITLE-ABS-KEY(tens) OR TITLE-ABS-KEY(pens) OR TITLE-ABS-KEY(dry needling) AND TITLE-ABS-KEY(bdnf) OR TITLE-ABS-KEY(brain derived neurotrophic factor) AND TITLE-ABS-KEY(clinical trials) AND NOT TITLE-ABS-KEY(animals))

Wos

(((((((((((((((((((((((((((((((((((((((ALL=(Pain)) OR ALL=(Chronic pain)) OR ALL=(musculoskeletal pain)) OR ALL=(musculoskeletal disorder)) OR ALL=(fibromyalgia)) OR ALL=(chronic fatigue syndrome)) OR ALL=(osteoarthritis)) OR ALL=(headache disorders)) OR ALL=(central sensitization)) OR ALL=(descending inhibition)) OR ALL=(long term potentiation)) OR ALL=(pain threshold)) OR ALL=(pain sensitivity)) OR ALL=(pain tolerance)) OR ALL=(pain perception)) AND ALL=(physical therapy modalities)) OR ALL=(physical therapy)) OR ALL=(physiotherapy)) OR ALL=(rehabilitation)) OR ALL=(exercise)) OR ALL=(pain education)) OR ALL=(pain neuroscience education)) OR ALL=(motor imagery)) OR ALL=(guided imagery)) OR ALL=(mental practice)) OR ALL=(mental imagery)) OR ALL=(Electrotherapy)) OR ALL=(electrical stimulation)) OR ALL=(tdcs)) OR ALL=(transcranial direct current stimulation)) OR ALL=(tms)) OR ALL=(transcranial magnetic stimulation)) OR ALL=(tens)) OR ALL=(pens)) OR ALL=(dry needling)) AND ALL=(bdnf)) OR ALL=(brain derived neurotrophic factor)) NOT ALL=(animals)) AND ALL=(clinical trial)

Embase

((((((((((((((((((((((((((((((((((((((ALL=(Pain)) OR ALL=(Chronic pain)) OR ALL=(musculoskeletal pain)) OR ALL=(musculoskeletal disorder)) OR ALL=(fibromyalgia)) OR ALL=(chronic fatigue syndrome)) OR ALL=(osteoarthritis)) OR ALL=(headache disorders)) OR ALL=(central sensitization)) OR ALL=(descending inhibition)) OR ALL=(long term potentiation)) OR ALL=(pain threshold)) OR ALL=(pain sensitivity)) OR ALL=(pain tolerance)) OR ALL=(pain perception)) AND ALL=(physical therapy modalities)) OR ALL=(physical therapy)) OR ALL=(physiotherapy)) OR ALL=(rehabilitation)) OR ALL=(exercise)) OR ALL=(pain education)) OR ALL=(pain neuroscience education)) OR ALL=(motor imagery)) OR ALL=(guided imagery)) OR ALL=(mental practice)) OR ALL=(mental imagery)) OR ALL=(Electrotherapy)) OR ALL=(electrical stimulation)) OR ALL=(tdcs)) OR ALL=(transcranial direct current stimulation)) OR ALL=(tms)) OR ALL=(transcranial magnetic stimulation)) OR ALL=(tens)) OR ALL=(pens)) OR ALL=(dry needling)) AND ALL=(bdnf)) OR ALL=(brain derived neurotrophic factor)) NOT ALL=(animals)) AND ALL=(clinical trial)
